# Is there a Relation between *Chlamydia* Infection and Primary Biliary Cirrhosis?

**DOI:** 10.1080/10446670310001642429

**Published:** 2003

**Authors:** Patrick S. C. Leung, Ogyi Park, Shuji Matsumura, Aftab A. Ansari, Ross L. Coppel, M. Eric Gershwin

**Affiliations:** 1 Division of Rheumatology Allergy and Clinical Immunology University of California Davis CA 95616 USA; 2 Department of Pathology Emory University School of Medicine Atlanta 30322 Georgia; 3 Department of Microbiology Monash University Clayton Victoria 3168 Australia

## Abstract

Over the past two decades, a number of studies have failed to provide direct evidence of specific microbial chronic infection in primary biliary cirrhosis (PBC). However, a recent report suggests that there is a specific association of *Chlamydia pneumoniae* in patients with PBC and that *C. pneumoniae* or similar antigens might play a role in the pathogenesis of disease. To determine if *Chlamydia* infection is associated with PBC, we applied a combination of immunological and molecular approaches to investigate (a) the serological reactivity against two common *Chlamydia* human pathogens, *C. pneumoniae* and *C. trachomatis*, by immunoblotting, (b) the presence of *Chlamydia* in liver samples of patients with PBC and controls by PCR amplification of *Chlamydia* specific 16S rRNA and (c) the presence of *Chlamydia* proteins in liver samples of patients with PBC and controls by immunohistochemical staining. By immunoblotting, *C. trachomatis* and *C. pneumoniae* specific serological antibodies were found in 52/57 (91.2%) AMA positive PBC, 7/33 (21/2%) of AMA negative PBC, 1/25 (4%) PSC, 0/15 (0%) Sjorgen's syndrome and 0/20 (0%) systemic lupus erythematosus patients and 0/20 (0%) healthy volunteers at 1:200 sera dilution. PBC sera reacted to *Chlamydia* and *E. coli* lysates in western blots up to a maximum of 10^-4^ dilution. However, PCR amplification of the *Chlamydia* specific 16S rRNA gene was negative in 25/25 PBC livers but positive in 1/4 PSC liver, 3/6 in other liver disease controls and 1/4 normal liver samples. While two commercially available specific monoclonal antibodies stained positive controls (*Chlamydia* infected HEp-2 cells) they failed to detect *Chlamydia* antigens in PBC livers. The detection of *Chlamydia* specific antibodies but not *Chlamydia* rRNA gene and *Chlamydia* antigens in PBC suggests that *Chlamydia* infection is not involved in PBC.


*Clinical & Developmental Immunology*, June–December 2003 VOL. 10 (2–4), pp. 227–233

Taylor & Francis healthsciences

### Is there a Relation between *Chlamydia* Infection and Primary Biliary Cirrhosis?

PATRICK S.C. LEUNG[a,*], OGYI PARK[a], SHUJI MATSUMURA[a], AFTAB A. ANSARI[b], ROSS L. COPPEL[c] and M. ERIC GERSHWIN[a]

[a]*Division of Rheumatology, Allergy and Clinical Immunology, University of California, Davis, CA 95616, USA;* [b]*Department of Pathology, Emory University School of Medicine, Atlanta 30322, Georgia;* [c]*Department of Microbiology, Monash University, Clayton, Victoria 3168, Australia*

Over the past two decades, a number of studies have failed to provide direct evidence of specific microbial chronic infection in primary biliary cirrhosis (PBC). However, a recent report suggests that there is a specific association of *Chlamydia pneumoniae* in patients with PBC and that *C. pneumoniae* or similar antigens might play a role in the pathogenesis of disease. To determine if *Chlamydia* infection is associated with PBC, we applied a combination of immunological and molecular approaches to investigate (a) the serological reactivity against two common *Chlamydia* human pathogens, *C. pneumoniae* and *C. trachomatis,* by immunoblotting, (b) the presence of *Chlamydia* in liver samples of patients with PBC and controls by PCR amplification of *Chlamydia* specific 16S rRNA and (c) the presence of *Chlamydia* proteins in liver samples of patients with PBC and controls by immunohistochemical staining. By immunoblotting, *C. trachomatis* and *C. pneumoniae* specific serological antibodies were found in 52/57 (91.2%) AMA positive PBC, 7/33 (21/2%) of AMA negative PBC, 1/25 (4%) PSC, 0/15 (0%) Sjorgen's syndrome and 0/20 (0%) systemic lupus erythematosus patients and 0/20 (0%) healthy volunteers at 1:200 sera dilution. PBC sera reacted to *Chlamydia* and *E. coli* lysates in western blots up to a maximum of $10^{-4}$ dilution. However, PCR amplification of the *Chlamydia* specific 16S rRNA gene was negative in 25/25 PBC livers but positive in 1/4 PSC liver, 3/6 in other liver disease controls and 1/4 normal liver samples. While two commercially available specific monoclonal antibodies stained positive controls (*Chlamydia* infected HEp-2 cells) they failed to detect *Chlamydia* antigens in PBC livers. The detection of *Chlamydia* specific antibodies but not *Chlamydia* rRNA gene and *Chlamydia* antigens in PBC suggests that *Chlamydia* infection is not involved in PBC.

*Keywords*: *Chlamydia*; Cross reactivity; Infection; Primary biliary cirrhosis

### INTRODUCTION

The breaking of tolerance by molecular mimicry of self-antigens by microbial proteins is an attractive hypothesis in the etiology of primary biliary cirrhosis (PBC) (Bjorkland and Totterman, 1994; Baum, 1995; Sutton and Neuberger, 2002; Datta, 2003; Hayakawa *et al.*, 2003; Plotz, 2003). PBC is serologically characterized by high titer anti-mitochondrial antibodies (AMA) in 95% of patients (Gershwin *et al.*, 2000). AMA are directed against E2 subunits of the 2- oxo-acid dehydrogenase complexes (2-OADC), and also against subunits E1 alpha, E1 beta and E3 binding protein of the pyruvate dehydrogenase complex (Leung *et al.*, 1997; Dubel *et al.*, 1999). AMA of PBC patients also react with bacterial E2s (Gershwin *et al.*, 2000). Reactivities are primarily peptide-specific but cross-reactivity between mitochondrial and microbial E2 antigens of respective complexes have been reported (Fussey *et al.*, 1990). The AMA immunodominant epitopes include the conserved sequence flanking the site of lipoyl attachment site of the 2-OADC.

It has been postulated that the initial stimulus for antibody production is chronic urinary tract infection (Baum, 1995). A number of studies have demonstrated significant bacteriuria in patients with PBC, with *E. coli* as the most common urinary isolate (Burroughs *et al.*, 1984; Rabinovitz *et al.*, 1992; Butler *et al.*, 1993; O'Donohue *et al.*, 1997). For example, *E. coli* rough forms were found in the urine of patients with significant bacteriuria including 39% in patients with PBC, 5.3% in patients with chronic liver disease and 41% in patients from the recurrent urinary tract infection group. Cross-reactivity between AMA and corresponding antigenic bands of *E. coli* has been demonstrated in sera from patients with PBC suggesting that AMA arise in "normal" women with recurrent bacteriuria and in females with PBC (Butler *et al.*, 1993).

*Corresponding author. Tel.: +1-530-752-2884. Fax: +1-530-754-6047. E-mail: psleung@ucdavis.edu

ISSN 1740-2522 print/ISSN 1740-2530 online © 2003 Taylor & Francis Ltd
DOI: 10.1080/10446670310001642429

Other studies have sought to associate microbial agents responsible for breaking the immune tolerance with mitochondrial proteins. For example, Xu *et al.* reported the evidence of human betaretrovirus infection in lymph nodes of patients with PBC by RT-PCR and immunohistochemistry (Xu *et al.*, 2003). Screening of random peptide libraries with a PDC-E2 specific monoclonal antibody yielded eight different peptide sequences with little consensus (Cha *et al.*, 1996). Tanaka *et al.* concluded that no consensus 16S rRNA microbial sequence could be found in PBC liver specimens by PCR amplification from liver tissue specimens from patients with PBC and non-PBC controls (Tanaka *et al.*, 1999). Altogether, these approaches, although rigorous could not identify any infectious agent specific associated with PBC.

Recently, it has been suggested *Chlamydia pneumoniae* or similar antigens play an important role in the pathogenesis of PBC (Abdulkarim *et al.*, 2002a,b). Briefly, *C. pneunomiae* antigens were detected in explant liver specimens from 25/25 patients with PBC (2 of whom were antimitochondrial antibody-negative) by immunohistochemistry. *C. pneumoniae* antigens were detected in periportal and lobular hepatocytes of PBC livers, whereas only 9/105 (8.5%) of patients in other categories (primary sclerosing cholangitis (PSC), alcoholic liver disease and chronic hepatitis C combined) were positive ($p < 0.01$, Fisher's exact T test). To verify that *Chlamydia* infection is associated with PBC, we have probed the antibody reactivities of PBC and control sera samples against two common *Chlamydia* species; *C. pneumoniae* and *C. trachomatis*, pursued PCR based molecular detection in liver samples of control and PBC patients of *Chlamydia* specific 16S rRNA gene and sought to demonstrate the presence of the two *Chlamydia* types by immunohistochemistry in liver samples from PBC patients and controls.

### MATERIALS AND METHODS

### Source of Antibodies

A total of 170 sera samples were used in this study. These include sera from patients with PBC ($n = 90$), PSC ($n = 25$), systemic lupus erythematosus ($n = 20$), Sjogren's syndrome ($n = 15$) and healthy volunteers ($n = 20$). All patients were diagnosed according to established criteria (Kaplan, 1996; Venables, 1998; Lee and Kaplan, 1999; Lee and Kaplan, 2002). In addition the sera from patients with PBC have been tested for the presence of AMA as described (Tanaka *et al.*, 2002), which revealed 57 AMA positive and 33 AMA negative sera. Anti-lipoic acid antibody was generated as described (Humphries and Szweda, 1998). Anti-*Chlamydia* antibodies were obtained from Virostat (Portland, ME). Monoclonal antibodies against 2-OADC-E2 were generated by immunization of Balb c mice with a recombinant triple hybrid containing the AMA epitopes of PDC-E2, BCOADC-E2 and OGDC-E2 (Migliaccio *et al.*, 1998).

### Tissue Samples

Explant liver specimens from 25 patients with PBC and 36 controls were studied. The PBC livers included 5 patients classified as stage I and II and 20 patients classified as stage III and IV. Controls included livers from patients with PSC ($n = 10$), other liver diseases including hepatitis ($n = 6$), cryptic cirrhosis ($n = 8$) and alcoholic liver disease ($n = 8$) as well as normal liver samples ($n = 4$).

### Preparation of Recombinant Proteins

Recombinant peptides of mammalian PDC-E2, BCOADC-E2, OGDC-E2 were purified from the corresponding expression clone in pGEX (Moteki *et al.*, 1996). Briefly, the *E coli* clone was grown in LB containing 50 μg/ml of ampicillin and induced with 1 mM isopropyl beta-D-thiogalactoside (Alexus, San Diego, CA) for 6 h. Cells were harvested and recombinant proteins were purified using glutathione agarose (Sigma) as described (Moteki *et al.*, 1996). An irrelevant expression protein of the shrimp tropomyosin in pGEX (Leung *et al.*, 1994) was purified similarly.

### Serological Immunoreactivity against *Chlamydia* by Immunoblotting

*Chlamydia trachomatis* elementary bodies were obtained from Advanced Biotechnologies (Columbia, Maryland). *C. pneumoniae* was prepared from HEp-2 cells culture as described (Tjhie *et al.*, 1997). A measured quantity of 100 μg of *Chlamydia* lysate was resolved on 10% SDS-polyacrylamide gel electrophoresis (SDA-PAGE) and transferred to nitrocellulose membrane. The membrane was then cut into 3 mm strips, blocked with 3% non-fat dry milk in phosphate buffered saline (PBS) for 1 h and then incubated with human sera (1:200 dilution) for 1 h. Membranes are then washed with PBS containing 0.05% tween 20 for 4 times, 10 min each before incubating with horseradish peroxidase conjugated anti-human Ig (Zymed, South San Francisco, CA for 1 h at room temperature. Membranes were then washed with PBS containing 0.05% tween 20. Antibody binding was visualized by incubating with a 0.05% solution of 3, 3′ diamino-benzidine (Sigma) and 0.01% hydrogen peroxide. To determine the identity of the *Chlamydia* immunoreactive bands, PBC sera (1:200 dilutions) were preincuabted with 100 μg of recombinant proteins of PDC-E2, BCOADC-E2 and OGDC-E2 and the irrelevant antigen noted above at 4°C for overnight prior to probing against *Chlamydia* by immunoblotting. In addition, 100 μg of *Chlamydia* lysate and the same amount of *E. coli* lysate were resolved by SDS-PAGE, and transferred onto nitrocellulose membrane.

Reactivity of PBC at $10^3 - 10^6$ of sera dilutions were analyzed by immunoblotting against equal concentrations of *Chlamydia* and *E. coli* lysate followed by chemiluminescent detection (Pierce, Rockford, IL).

### PCR Amplification of *Chlamydia* 16S rRNA Gene

Total DNA was isolated from explant liver samples using the QIAmp DNA mini-kit (Qiagen, Valencia, CA) according to manufacturer's instruction. Touch down polymerase chain reaction (PCR) was conducted to amplify the 16S rRNA gene of *C. pneumoniae* from liver DNA samples using specific primers (CpnA): 5′-TGACAACTGTAGAAATACAGC-3′ and (CpnB): 5′-CGCCTCTCTCCTATAAAT-3′ as described (Gaydos *et al.*, 1992; Gaydos *et al.*, 1994a,b; Boman *et al.*, 1999). Similarly, the *C. trachomatis* 16S rRNA gene was amplified using specific primers (CtnA): 5′-TGACCGCGGCAGAAATGTCGT-3′ and (CtnB) 5′-CGCCTCTCTCCCTTGCGG-3′ as described (Gaydos *et al.*, 1992; Gaydos *et al.*, 1994a,b; Boman *et al.*, 1999). *C. pneumoniae* and *C. trachomatis* DNA were included as controls. Briefly, the reaction mixture contained liver DNA samples, 0.5 μM primers, 0.2 mM dNTPs, 1 × PCR buffer and 1 unit of Taq polymerase. Samples were subjected to 6 cycles of denaturation (94°C, 1 min), annealing (60–54°C, 1°C step down each cycle, 1 min) and extension (72°C, 1 min), followed by 30 cycles of denaturation (94°C, 1 min), annealing (55°C, 1 min) and extension (72°C, 1 min). Negative (DNAse, RNAse free water and DNA extracted from *E. coli*) and positive controls (DNA extracted from *C. pneumoniae* and *C. trachomatis*) were included. PCR products were resolved by agarose gel electrophoresis and visualized by ethidium bromide staining (0.5 μg/ml). Positive and negative controls are included throughout (Boman *et al.*, 1999).

### Immunohistochemical Staining

Immunohistochemical staining with monoclonal antibodies against *C. pneumoniae* were performed on liver sections from patients with PBC ($n = 10$) and other disease controls (six PSC, one alcoholic liver cirrhosis, five cryptic cirrhosis and six Non-A, Non-B hepatic cirrhosis). Briefly, paraffin sections were incubated at 65°C for overnight and deparaffinized in four changes of xylene. The slides were then washed in two 100% alcohol and two 95% alcohol rinses and then finally rinsed in tap water. The slides were washed in phosphate buffered saline (PBS, pH 7.4) and incubated with blocking solution (1.5% of normal horse serum in PBS). Following blocking, slides were incubated with 1:20 diluted monoclonal antibody against *C. pneumoniae* (Virostat, Portland, ME) for 1 h in a humidified chamber and thereafter washed three times in PBS. After that, the slides were incubated with biotin labelled anti-mouse-IgG for 30 min and washed three times in PBS before incubating with avidin and biotinylated HRP complex (Vector Laboratories, Burlingame, CA) solution. Reactivity was visualized by the addition of alkaline phosphatase substrate. (The slides were mounted with crystal mounting medium and analyzed by fluorescent microscopy using the Olympus Provis Microscope (Olympus America, Melille, NY).

### RESULTS

### Serological Immunoreactivity against *Chlamydia*

By immunoblotting, *Chlamydia* specific serological antibodies were found in 52/57 (91.2%) AMA positive PBC and 7/33 (21.2%) of AMA negative PBC sera. Out of the 90 PBC sera, 59(65.6%) reacted with *C. trachomatis* and 51(56.7%) reacted with *C. pneumoniae*. 1/25 (4%) of PSC sera reacted to both *C. trachomatis* and *C. pneumoniae,* 0/15 (0%) of Sjorgen syndrome and 0/20 (0%) of systemic lupus erythematosus patients and 0/20 (0%) of healthy subjects reacted to either of the *Chlamydia* species (Table I). Moreover, the identities of the major *Chlamydia* reactive proteins were determined as the E2 subunits of 2-oxo-acid dehydrogenase complex by the antigen inhibition studies and confirmed by their specific reactivities to monoclonal antibodies to mammalian 2-OADCC- E2 (Fig. 1). Irrelevant recombinant protein of the shrimp tropomyosin did not inhibit.

TABLE I Serological reactivity to *Chlamydia* by immunoblotting

| *Chlamydia* species | Group (No. of samples) | No. of positive samples/total (%) |
|---|---|---|
| *Chlamydia trachomatis* | Primary biliary cirrhosis ($n = 90$) | 59/90 (65.6%) |
| | Primary sclerosing cholangitiss ($n = 25$) | 1/25 (4%) |
| | Sjogren's syndrome ($n = 15$) | 0/15 (0%) |
| | Systemic lupus erythematosus ($n = 20$) | 0/20 (0%) |
| | Normal ($n = 20$) | 0/20 (0%) |
| *Chlamydia pneumoniae* | Primary biliary cirrhosis ($n = 90$) | 51/90 (56.7%) |
| | Primary sclerosing cholangitis ($n = 25$) | 1/25 (4%) |
| | Sjogren's syndrome ($n = 15$) | 0/15 (0%) |
| | Systemic lupus erythematosus ($n = 20$) | 0/20 (0%) |
| | Normal ($n = 20$) | 0/20 (0%) |

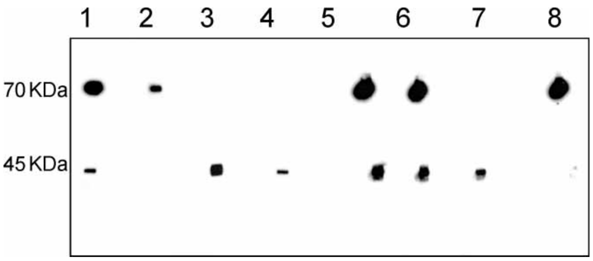

FIGURE 1 Immunoreactivity of *Chlamydia pneumoniae* lysate. Note the reactivity with anti-lipoic acid antibody (lane 1), monoclonal antibody to PDC-E2 (lane 2); to OGDC-E2 (lane 3); to BCOADC-E2 (lane 4); Sera (1:1000) from patients with PBC (lane 5); Sera from patients with PBC after absorption with 3% non-fat dry milk in PBS (lane 6) and the lost of reactivity to a 70 KDa band when PBC serum is first absorbed with recombinant PDC-E2 (lane 7); lost of reactivity to a 45 KDa band when PBC serum is first absorbed with recombinant OGDC-E2 (lane 8).

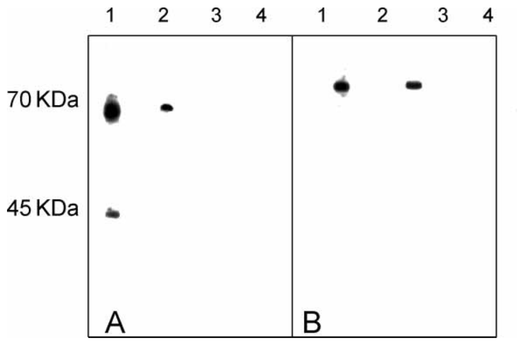

FIGURE 2 Lysates of *E. coli* (A) and *C. pneumoniae* (B) were separated by SDS-PAGE, transferred to nitrocellulose and probed with a representative serum from a AMA positive patient with PBC at serial dilutions of $10^{-3}$–$10^{-6}$. Note the reactivity to both: *E. coli* lysate (A) and *C. pneumoniae* lysate (B) at sera dilutions of $10^{-3}$ and $10^{-4}$ (lanes 1 and 2) but not at $10^{-5}$ and $10^{-6}$ sera dilution (lanes 3 and 4).

### Detection of *C. trachomatis* and *C. pneumoniae* Specific 16S rRNA Gene

PCR amplification of *C. trachomatis* and *C. pneumoniae* specific16S rRNA gene showed that neither *Chlamydia* 16S rRNA gene was present in livers from patients with PBC, but was present in low percentage of the other controls (Table II).

### Immunohistochemical Detection of *Chlamydia* in Liver Samples

*C. pneumoniae* were not detected on the bile ducts and hepatocytes of the liver sections from 10 PBC patients as well as 18 livers sections from other disease controls.

### Titer of PBC Patient Sera against *Chlamydia* and *E. coli*

PBC sera reacted to both *Chlamydia* and *E. coli* at $10^{-3}$ and $10^{-4}$ sera dilution but did not react at sera dilution greater than $10^{4}$(Fig. 2).

### DISCUSSION

The underlying mechanism(s) in the breaking of immune tolerance is one of the most important issues in the etiopathology of autoimmune diseases. One hypothesis is based on molecular mimicry, i.e. mimicry by infectious agents of host antigens may induce cross-reactive autoimmune responses to epitopes within host proteins which, in susceptible individuals, may tip the balance towards mounting an immunological response and subsequently lead to autoimmune disease (Davies, 1997). In fact, a number of reports support this hypothesis (Zhao *et al.*, 1998; Fournel and Muller, 2002). It has been suggested that infection can probably cause T cell activation in multiple sclerosis (MS) susceptible individuals (Hafler, 1999) and several microbial agents including *C. pneumoniae* have been implicated as the intracellular pathogens causing MS (Hunter and Hafler, 2000). On the other hand, a search for molecular mimicry in PBC has been done using monoclonal antibodies to PDC-E2 and random peptide libraries (Cha *et al.*, 1996; Leung *et al.*, 1996) and found mimeotope peptides that did not show any significant sequence homology to any known microbial peptides and did not generate PBC in animals.

TABLE II PCR amplification of *Chlamydia* 16S rRNA gene

| Group | No. of samples tested | No. positive to *C. pneumoniae* | No. positive to *C. trachomatis* |
|---|---|---|---|
| Primary biliary cirrhosis | 25 | 0/25 | 0/25 |
| Primary sclerosing cholangitis | 10 | 1/10 | 1/10 |
| Hepatitis | 6 | 1/6 | 0/6 |
| Cryptic cirrhosis | 8 | 3/8 | 3/8 |
| Alcoholic liver disease | 8 | 2/8 | 1/8 |
| Normal | 4 | 1/4 | 1/4 |

Based on the significant sequence homology between the AMA epitopes and the *E. coli* lipoyl enzymes, it was hypothesized that the initial stimulus for AMA production may result from chronic urinary tract infection (Baum, 1995). Although AMA themselves are not pathogenic, antigen specific CD4+ T cells could be primed, recognizing the lipoyl domain epitope in association with class II HLA and initiate the autoimmune cascade in patients with PBC. The association of urinary tract infections in patients with PBC has been studied and the data are inconclusive (Burroughs *et al.*, 1984; Rabinovitz *et al.*, 1992; Butler *et al.*, 1993; O'Donohue *et al.*, 1997). Similarly, data on association of PBC with other microbial agents remain elusive (Tanaka *et al.*, 1999; Heathcote, 2000; Samonakis *et al.*, 2003). We have used a three prone approach to determine if *Chlamydia* infection is involved in the pathogenesis of PBC through the use of a combination of immunological and molecular approaches.

*Chlamydia* are obligate intracellular eubacteria which are phylogenetically distinct from other bacteria. Two well-known *Chlamydia* human pathogens are

*C. pneumoniae* and *C. trachomatis*. *C. pneumoniae* is a widespread pathogen of humans causing pneumonia and bronchitis and it accounts for approximately 10% of pneumonia and 5% of bronchitis cases in the United States. In addition, there are reports associating *C. pneumoniae* infection with atherosclerosis and abdominal aortic aneurysm (Danesh *et al.*, 1997; Juvonen *et al.*, 1997a; Juvonen *et al.*, 1997b). *C. trachomatis* infection causes trachoma, an ocular infection that leads to blindness and sexually transmitted diseases (Tabbara, 2001; Gaynor *et al.*, 2003; Jensen *et al.*, 2003). Analysis of the *C. pneumoniae* and *C. trachomatis* genome (Kalman *et al.*, 1999) showed that the two genomes have a low level of DNA homology to the mammalian genome. However, genes for central metabolic pathways, including aerobic respiration are present and electron transport may be supported by pyruvate. Thus, it is not surprising that lipoyl containing proteins, which are necessary in mammalian aerobic respiration, are detectable in *Chlamydia* when anti-lipoic acid antibody was used as a probe (Fig. 1). Furthermore, the *Chlamydia* lipoic acid containing proteins are recognized by AMA and the Ig reactivity can be inhibited by absorption with recombinant E2 proteins of the 2-OADC pathway (Fig. 1), suggesting that AMA in PBC are binding to the lipoyl domains of the *Chlamydia* homologue of the mammalian respiratory enzymes. 65.6% of the PBC sera sample tested reacted positively to *Chlamydia*, which is similar to the prevalence of antibodies to *C. pneumoniae* (40–60%) in the general population (Cook and Honeybourne, 1995; Niki and Kishimoto, 1996). It is interesting to note that a surprisingly low percentage of control sera reacted positively to either *C. pneumoniae* or *C. trachomatis*. On the other hand, 70% of the PBC sera, were positive to *C. trachomatis*. When the titer of PBC sera against *Chlamydia* vs. against *E. coli*, PBC sera reacted to both *E. coli* and *Chlamydia* at sera dilutions up to $10^4$ as detected by chemiluminescence. This modest titer suggests that the serological antibody reactivity to *Chlamydia* is at best comparable to other common human microbes.

PCR is a highly sensitive and specific method for detection and quantification of specific nucleic acids and its application to the detection of slow growing microorganisms in autoimmune diseases has been recently reviewed (Cuchacovich *et al.*, 2003). *C. pneumoniae* and *C. trachomatis* specific 16S rRNA was not detected in any of the PBC liver samples but present in low percentages in the other liver disease control and normal volunteers. Due to the ubiquitous nature of *Chlamydia*, special precautions were specifically used to avoid not only false positive and but care was taken to avoid generating false negative results in the detection of *Chlamydia* by PCR. Thus, the absence of any *Chlamydia* specific 16S rRNA gene in PBC livers seems unlikely to be due to experimental or technical issues such as DNA quality, primer specificities and reaction conditions. Of note, *C. pneumoniae* has been reported to have a strong association with patients with MS, but a recent report using a large population of MS patients and controls was unable to confirm the increase presence of *C. pneumoniae* genome in the spinal fluid of MS patients (Boman *et al.*, 2000; Krametter *et al.*, 2001). Furthermore, a surprisingly low prevalence of *C. trachomatis* infection was reported in the three studies using PCR (1.06, 0 and 1.48%, respectively) giving an overall prevalence of 0.98% (Andreu Domingo *et al.*, 2002). Thus the detection of *Chlamydia* specific antibodies but not *Chlamydia* rRNA gene and *Chlamydia* antigens in PBC suggests that *Chlamydia* infection is not involved in PBC.

Despite multiple and rigorous studies, the linkage between microbial infections and autoimmune diseases remains still elusive. The apparent lack of involvement of *Chlamydia* infection in PBC as reported here suggests that the molecular mimicry hypothesis in PBC may require modification. Recently, the hypothesis that PBC may be due to an initial insult with an environmental agent, possibly as a xenobiotic agent has been explored (Sasaki *et al.*, 2000; Long *et al.*, 2001). Since optimal reactivity of AMA requires the lipoyl domain as a component of the autoantigen (Migliaccio *et al.*, 2001), its structural configuration has been reasoned to be important for the breakdown of self tolerance in PBC. Hence, exposure of genetically susceptible individuals to a xenobiotic agent, which mimics the lipoylated peptide of PDC-E2, may be important in the etiology of PBC. In a recent study, we synthesized the immunodominant 12 amino acid peptide epitope within the inner lipoyl domain of PDC-E2 and replaced the lipoic acid moiety with a battery of synthetic structures designed to mimic a xenobiotically-modified lipoyl hapten, and quantified the reactivities of these structures with sera from PBC patients. Particularly noteworthy was our finding that AMA from all patients with PBC, but not controls, reacted against several of the organic modified mimetopes as well as, or significantly better than, to the native lipoyl domain (Long *et al.*, 2001). This is further supported by the recent data that rabbits immunized by one of such xenobiotic agents, 6-bromohexanote induced the production of AMA (Leung *et al.*, 2003). Most recently, a specific association of a unique xenobiotic metabolizing bacteria *Novosphingobium aromaticivroans* (Takeuchi *et al.*, 2001; Pinyakong *et al.*, 2003) and PBC has been reported (Selmi *et al.*, 2003). *N. aromaticivorans* is ubiquitous in the environment (Takeuchi *et al.*, 2001; Fujii *et al.*, 2002; Tiirola *et al.*, 2002; Fujii *et al.*, 2003) and has the highest homology in the lipoyl domains between two of its lipoylated proteins and the human PDC-E2 in the known microbial world. Moreover, 100% (77/77) of anti-PDC-E2 positive and 12% (2/17) of AMA negative sera from patients with PBC recognize these two lipoyl containing proteins, while no such reactivity was detected in 200 control sera. Antibody titers to these *N. aromaticivorans* lipoyl-containing proteins was detectable even at dilutions of 1:1,000,000 and were up to 1000-fold stronger than the titers for *E. coli* lipoylated proteins. In addition to the reactivity towards these two lipoylated proteins, sera from patients with PBC also recognize other non-lipoylated *N. aromaticivorans* proteins. Moreover, PCR amplification of the *N. aromaticivorans* 16S rRNA gene from fecal samples showed that *N. aromaticivorans* is present in approximately 25% of PBC patients and controls. Altogether, these data the suggests that xenobiotic modification of lipoyl moiety of the *N. aromaticivorans* PDC-E2 homologues as a possible mechanism in the etiology of PBC. The origin of autoimmunity is clearly multifactorial and molecular mimicry of infectious agent itself cannot fully account for the specificities and spectrum of autoimmune diseases. The role of xenobiotics, in conjunction with presence of xenobiotic metabolizing microorganisms and the hypothesis of molecular mimicry in the induction of autoimmune diseases (Pumford and Halmes, 1997) warrant further investigation.

### *Acknowledgements*

This work was supported in part by NIH grant AI 39558.

### *References*

Abdulkarim, A., Petrovic, L., Kim, R., Angulo, P., Keack, J. and Lindor, K. (2002a) "Primary biliary cirrhosis: an infectious disease caused by *Chlamydia pneumoniae*?", *J. Hepatol.* **36**(Suppl 1), 152.

Abdulkarim, A.S., Petrovic, L.M., Kim, W.R., Angulo, P.G., Keach, J.C. and Lindor, K.D. (2002b) "Primary biliary cirrhosis: an infectious disease caused by *Chlamydia pneumoniae*?", *Gastroenterology*, 122.

Andreu Domingo, A., Pumarola Sune, T., Sanz Colomo, B., Sobejano Garcia, L., Xercavins Montosa, J., Coll Escursell, O., Lopez Lopez, M.A. and Codina Grau, G. (2002) "[Prevalence of Chlamydia trachomatis infection, as evaluated by molecular biology methods]", *Enferm. Infect. Microbiol. Clin.* **20**, 205–207.

Baum, H. (1995) "Mitochondrial antigens, molecular mimicry and autoimmune disease", *Biochim. Biophys. Acta* **1271**, 111–121.

Bjorkland, A. and Totterman, T.H. (1994) "Is primary biliary cirrhosis an autoimmune disease?", *Scand. J. Gastroenterol, Suppl.* **204**, 32–39.

Boman, J., Gaydos, C.A. and Quinn, T.C. (1999) "Molecular diagnosis of Chlamydia pneumoniae infection", *J. Clin. Microbiol.* **37**, 3791–3799.

Boman, J., Roblin, P.M., Sundstrom, P., Sandstrom, M. and Hammerschlag, M.R. (2000) "Failure to detect *Chlamydia pneumoniae* in the central nervous system of patients with MS", *Neurology* **54**, 265.

Burroughs, A.K., Rosenstein, I.J., Epstein, O., Hamilton-Miller, J.M., Brumfitt, W. and Sherlock, S. (1984) "Bacteriuria and primary biliary cirrhosis", *Gut* **25**, 133–137.

Butler, P., Valle, F., Hamilton-Miller, J.M., Brumfitt, W., Baum, H. and Burroughs, A.K. (1993) "M2 mitochondrial antibodies and urinary rough mutant bacteria in patients with primary biliary cirrhosis and in patients with recurrent bacteriuria", *J. Hepatol.* **17**, 408–414.

Cha, S., Leung, P.S., van de Water, J., Tsuneyama, K., Joplin, R.E., Ansari, A.A., Nakanuma, Y., Schatz, P.J., Cwirla, S., Fabris, L.E., Neuberger, J.M., Gershwin, M.E. and Coppel, R.L. (1996) "Random phage mimotopes recognized by monoclonal antibodies against the pyruvate dehydrogenase complex-E2 (PDC-E2)", *Proc. Natl Acad. Sci. USA* **93**, 10949–10954.

Cook, P.J. and Honeybourne, D. (1995) "Clinical aspects of *Chlamydia pneumoniae* infection", *Presse Med.* **24**, 278–282.

Cuchacovich, R., Quinet, S. and Santos, A.M. (2003) "Applications of polymerase chain reaction in rheumatology", *Rheum. Dis. Clin. North Am.* **29**, 1–20.

Danesh, J., Collins, R. and Peto, R. (1997) "Chronic infections and coronary heart disease: is there a link?", *Lancet* **350**, 430–436.

Datta, S.K. (2003) "Major peptide autoepitopes for nucleosome-centered T and B cell interaction in human and murine lupus", *Ann. N Y Acad. Sci.* **987**, 79–90.

Davies, J.M. (1997) "Molecular mimicry: can epitope mimicry induce autoimmune disease?", *Immunol. Cell Biol.* **75**, 113–126.

Dubel, L., Tanaka, A., Leung, P.S., van de Water, J., Coppel, R., Roche, T., Johanet, C., Motokawa, Y., Ansari, A. and Gershwin, M.E. (1999) "Autoepitope mapping and reactivity of autoantibodies to the dihydrolipoamide dehydrogenase-binding protein (E3BP) and the glycine cleavage proteins in primary biliary cirrhosis", *Hepatology* **29**, 1013–1018.

Fournel, S. and Muller, S. (2002) "Peptides as DNA mimics: cross-reactivity and mimicry in systemic autoimmune diseases", *Cell Mol. Life Sci.* **59**, 1280–1284.

Fujii, K., Kikuchi, S., Satomi, M., Ushio-Sata, N. and Morita, N. (2002) "Degradation of 17beta-estradiol by a gram-negative bacterium isolated from activated sludge in a sewage treatment plant in Tokyo, Japan", *Appl. Environ. Microbiol.* **68**, 2057–2060.

Fujii, K., Satomi, M., Morita, N., Motomura, T., Tanaka, T. and Kikuchi, S. (2003) "Novosphingobium tardaugens sp. nov., an oestradiol-degrading bacterium isolated from activated sludge of a sewage treatment plant in Tokyo", *Int. J. Syst. Evol. Microbiol.* **53**, 47–52.

Fussey, S.P., Ali, S.T., Guest, J.R., James, O.F., Bassendine, M.F. and Yeaman, S.J. (1990) "Reactivity of primary biliary cirrhosis sera with Escherichia coli dihydrolipoamide acetyltransferase (E2p): characterization of the main immunogenic region", *Proc. Natl Acad. Sci. USA* **87**, 3987–3991.

Gaydos, C.A., Quinn, T.C. and Eiden, J.J. (1992) "Identification of Chlamydia pneumoniae by DNA amplification of the 16S rRNA gene", *J. Clin. Microbiol.* **30**, 796–800.

Gaydos, C.A., Eiden, J.J., Oldach, D., Mundy, L.M., Auwaerter, P., Warner, M.L., Vance, E., Burton, A.A. and Quinn, T.C. (1994a) "Diagnosis of Chlamydia pneumoniae infection in patients with community-acquired pneumonia by polymerase chain reaction enzyme immunoassay", *Clin. Infect. Dis.* **19**, 157–160.

Gaydos, C.A., Roblin, P.M., Hammerschlag, M.R., Hyman, C.L., Eiden, J.J., Schachter, J. and Quinn, T.C. (1994b) "Diagnostic utility of PCR-enzyme immunoassay, culture, and serology for detection of Chlamydia pneumoniae in symptomatic and asymptomatic patients", *J. Clin. Microbiol.* **32**, 903–905.

Gaynor, B.D., Miao, Y., Cevallos, V., Jha, H., Chaudary, J.S., Bhatta, R., Osaki-Holm, S., Yi, E., Schachter, J., Whitcher, J.P. and Lietman, T. (2003) "Eliminating trachoma in areas with limited disease", *Emerg. Infect. Dis.* **9**, 596–598.

Gershwin, M.E., Ansari, A.A., Mackay, I.R., Nakanuma, Y., Nishio, A., Rowley, M.J. and Coppel, R.L. (2000) "Primary biliary cirrhosis: an orchestrated immune response against epithelial cells", *Immunol. Rev.* **174**, 210–225.

Hafler, D.A. (1999) "The distinction blurs between an autoimmune versus microbial hypothesis in multiple sclerosis", *J. Clin. Investig.* **104**, 527–529.

Hayakawa, K., Asano, M., Shinton, S.A., Gui, M., Wen, L.J., Dashoff, J. and Hardy, R.R. (2003) "Positive selection of anti-thy-1 autoreactive B-1 cells and natural serum autoantibody production independent from bone marrow B cell development", *J. Exp. Med.* **197**, 87–99.

Heathcote, E.J. (2000) "Management of primary biliary cirrhosis. The american association for the study of liver diseases practice guidelines", *Hepatology* **31**, 1005–1013.

Humphries, K.M. and Szweda, L.I. (1998) "Selective inactivation of alpha-ketoglutarate dehydrogenase and pyruvate dehydrogenase: reaction of lipoic acid with 4-hydroxy-2-nonenal", *Biochemistry* **37**, 15835–15841.

Hunter, S.F. and Hafler, D.A. (2000) "Ubiquitous pathogens: links between infection and autoimmunity in MS?", *Neurology* **55**, 164–165.

Jensen, I.P., Fogh, H. and Prag, J. (2003) "Diagnosis of *Chlamydia trachomatis* infections in a sexually transmitted disease clinic: evaluation of a urine sample tested by enzyme immunoassay and polymerase chain reaction in comparison with a cervical and/or a urethral swab tested by culture and polymerase chain reaction", *Clin. Microbiol. Infect.* **9**, 194–201.

Juvonen, J., Juvonen, T., Laurila, A., Alakarppa, H., Lounatmaa, K., Surcel, H.M., Leinonen, M., Kairaluoma, M.I. and Saikku, P. (1997a) "Demonstration of Chlamydia pneumoniae in the walls of abdominal aortic aneurysms", *J. Vasc. Surg.* **25**, 499–505.

Juvonen, J., Laurila, A., Juvonen, T., Alakarppa, H., Surcel, H.M., Lounatmaa, K., Kuusisto, J. and Saikku, P. (1997b) "Detection of

*Chlamydia pneumoniae* in human nonrheumatic stenotic aortic valves", *J. Am. Coll. Cardiol.* **29**, 1054–1059.

Kalman, S., Mitchell, W., Marathe, R., Lammel, C., Fan, J., Hyman, R.W., Olinger, L., Grimwood, J., Davis, R.W. and Stephens, R.S. (1999) "Comparative genomes of *Chlamydia pneumoniae* and *C. trachomatis*", *Nat. Genet.* **21**, 385–389.

Kaplan, M.M. (1996) "Primary biliary cirrhosis", *N. Engl. J. Med.* **335**, 1570–1580.

Krametter, D., Niederwieser, G., Berghold, A., Birnbaum, G., Strasser-Fuchs, S., Hartung, H.P. and Archelos, J.J. (2001) "*Chlamydia pneumoniae* in multiple sclerosis: humoral immune responses in serum and cerebrospinal fluid and correlation with disease activity marker", *Mult. Scler.* **7**, 13–18.

Lee, Y.M. and Kaplan, M.M. (1999) "Treatment of primary biliary cirrhosis and primary sclerosing cholangitis: use of ursodeoxycholic acid", *Curr. Gastroenterol. Rep.* **1**, 38–41.

Lee, Y.M. and Kaplan, M.M. (2002) "Management of primary sclerosing cholangitis", *Am. J. Gastroenterol.* **97**, 528–534.

Leung, P.S., Chu, K.H., Chow, W.K., Ansari, A., Bandea, C.I., Kwan, H.S., Nagy, S.M. and Gershwin, M.E. (1994) "Cloning, expression, and primary structure of Metapenaeus ensis tropomyosin, the major heat-stable shrimp allergen", *J. Allergy Clin. Immunol.* **94**, 882–890.

Leung, P.S., Cha, S., Joplin, R.E., Galperin, C., van de Water, J., Ansari, A.A., Coppel, R.L., Schatz, P.J., Cwirla, S., Fabris, L.E., Neuberger, J.M. and Gershwin, M.E. (1996) "Inhibition of PDC-E2 human combinatorial autoantibodies by peptide mimotopes", *J. Autoimmun.* **9**, 785–793.

Leung, P.S., Coppel, R.L., Ansari, A., Munoz, S. and Gershwin, M.E. (1997) "Antimitochondrial antibodies in primary biliary cirrhosis", *Semin. Liver Dis.* **17**, 61–69.

Leung, P.S., Quan, C., Park, O., van de Water, J., Kurth, M.J., Nantz, M.H., Ansari, A.A., Coppel, R.L., Lam, K.S. and Gershwin, M.E. (2003) "Immunization with a xenobiotic 6-bromohexanoate bovine serum albumin conjugate induces antimitochondrial antibodies", *J. Immunol.* **170**, 5326–5332.

Long, S.A., Quan, C., van de Water, J., Nantz, M.H., Kurth, M.J., Barsky, D., Colvin, M.E., Lam, K.S., Coppel, R.L., Ansari, A. and Gershwin, M.E. (2001) "Immunoreactivity of organic mimeotopes of the E2 component of pyruvate dehydrogenase: connecting xenobiotics with primary biliary cirrhosis", *J. Immunol.* **167**, 2956–2963.

Migliaccio, C., Nishio, A., van de Water, J., Ansari, A.A., Leung, P.S., Nakanuma, Y., Coppel, R.L. and Gershwin, M.E. (1998) "Monoclonal antibodies to mitochondrial E2 components define autoepitopes in primary biliary cirrhosis", *J. Immunol.* **161**, 5157–5163.

Migliaccio, C., van de Water, J., Ansari, A.A., Kaplan, M.M., Coppel, R.L., Lam, K.S., Thompson, R.K., Stevenson, F. and Gershwin, M.E. (2001) "Heterogeneous response of antimitochondrial autoantibodies and bile duct apical staining monoclonal antibodies to pyruvate dehydrogenase complex E2: the molecule versus the mimic", *Hepatology* **33**, 792–801.

Moteki, S., Leung, P.S.C., Coppel, R.L., Dickson, E.R., Kaplan, M.M., Munoz, S. and Gershwin, M.E. (1996) "Use of designer triple expression hybrid clone for three different lipoyl domains for the detection of anti-mitochondrial antibodies", *Hepatology* **24**, 97–103.

Niki, Y. and Kishimoto, T. (1996) "Epidemiology of intracellular pathogens", *Clin. Microbiol. Infect.* **1**(Suppl 1), S11–S13.

O'Donohue, J., Workman, M.R., Rolando, N., Yates, M., Philpott-Howard, J. and Williams, R. (1997) "Urinary tract infections in primary biliary cirrhosis and other chronic liver diseases", *Eur. J. Clin. Microbiol. Infect. Dis.* **16**, 743–746.

Pinyakong, O., Habe, H. and Omori, T. (2003) "The unique aromatic catabolic genes in sphingomonads degrading polycyclic aromatic hydrocarbons (PAHs)", *J. Gen. Appl. Microbiol.* **49**, 1–19.

Plotz, P.H. (2003) "The autoantibody repertoire: searching for order", *Nat. Rev. Immunol.* **3**, 73–78.

Pumford, N.R. and Halmes, N.C. (1997) "Protein targets of xenobiotic reactive intermediates", *Annu. Rev. Pharmacol. Toxicol.* **37**, 91–117.

Rabinovitz, M., Prieto, M., Gavaler, J.S. and Van Thiel, D.H. (1992) "Bacteriuria in patients with cirrhosis", *J. Hepatol.* **16**, 73–76.

Samonakis, D.N., Chatzicostas, C., Vardas, E., Roussomoustakaki, M. and Kouroumalis, E.A. (2003) "Increased incidence of fungal infections in advanced primary biliary cirrhosis", *J. Clin. Gastroenterol.* **36**, 369.

Sasaki, M., Ansari, A., Pumford, N., van de Water, J., Leung, P.S., Humphries, K.M., Szweda, L.I., Nakanuma, Y., Roche, T.E., Coppel, R.L., Bach, J.F. and Gershwin, M.E. (2000) "Comparative immunoreactivity of anti-trifluoroacetyl (TFA) antibody and anti-lipoic acid antibody in primary biliary cirrhosis: searching for a mimic", *J. Autoimmun.* **15**, 51–60.

Selmi, C., Balkwill, D.L., Invernizzi, P., Ansari, A., Coppel, R.L., Podda, M., Leung, P.S. and Kenny, T.P. (2003) "Patients with PBC react against *Novosphingobium aromaticivorans*, a uniquitous xenobitic metabolizing bacteria", *Hepatology*, In Press.

Sutton, I. and Neuberger, J. (2002) "Primary biliary cirrhosis: seeking the silent partner of autoimmunity", *Gut* **50**, 743–746.

Tabbara, K.F. (2001) "Trachoma: a review", *J. Chemother.* **13**(Suppl 1), 18–22.

Takeuchi, M., Hamana, K. and Hiraishi, A. (2001) "Proposal of the genus *Sphingomonas sensu stricto* and three new genera, *Sphingobium, Novosphingobium* and *Sphingopyxis*, on the basis of phylogenetic and chemotaxonomic analyses", *Int. J. Syst. Evol. Microbiol.* **51**, 1405–1417.

Tanaka, A., Prindiville, T.P., Gish, R., Solnick, J.V., Coppel, R.L., Keeffe, E.B., Ansari, A. and Gershwin, M.E. (1999) "Are infectious agents involved in primary biliary cirrhosis? A PCR approach", *J. Hepatol.* **31**, 664–671.

Tanaka, A., Miyakawa, H., Luketic, V.A., Kaplan, M., Storch, W.B. and Gershwin, M.E. (2002) "The diagnostic value of anti-mitochondrial antibodies, especially in primary biliary cirrhosis", *Cell. Mol. Biol. (Noisy-le-grand)* **48**, 295–299.

Tiirola, M.A., Mannisto, M.K., Puhakka, J.A. and Kulomaa, M.S. (2002) "Isolation and characterization of *Novosphingobium sp.* strain MT1, a dominant polychlorophenol-degrading strain in a groundwater bioremediation system", *Appl. Environ. Microbiol.* **68**, 173–180.

Tjhie, J.H., Roosendaal, R., MacLaren, D.M. and Vandenbroucke-Grauls, C.M. (1997) "Improvement of growth of *Chlamydia pneumoniae* on HEp-2 cells by pretreatment with polyethylene glycol in combination with additional centrifugation and extension of culture time", *J. Clin. Microbiol.* **35**, 1883–1884.

Venables, P.J. (1998) "Undifferentiated connective tissue diseases: mixed or muddled?", *Lupus* **7**, 73–74.

Xu, L., Shen, Z., Guo, L., Fodera, B., Keogh, A., Joplin, R., O'Donnell, B., Aitken, J., Carman, W., Neuberger, J. and Mason, A. (2003) "Does a betaretrovirus infection trigger primary biliary cirrhosis?", *Proc. Natl Acad. Sci. USA* **100**, 8454–8459.

Zhao, Z.S., Granucci, F., Yeh, L., Schaffer, P.A. and Cantor, H. (1998) "Molecular mimicry by herpes simplex virus-type 1: autoimmune disease after viral infection", *Science* **279**, 1344–1347.

